# Strategic Thinking to Improve Surgical Training in the United Kingdom

**DOI:** 10.7759/cureus.4683

**Published:** 2019-05-16

**Authors:** Daniel L Ashmore

**Affiliations:** 1 General Surgery, Hull and East Yorkshire Hospitals National Health Service Trust, Hull, GBR

**Keywords:** surgical training, thinking

## Abstract

Surgical training in the United Kingdom (UK) is facing crucial challenges. Multiple fundamental changes in recent years have meant the same high-quality training needs to be delivered in a shorter duration. In this review, we consider the current training pathways for surgery in the UK, the impact of the European Working Time Directive (EWTD), the ongoing issue of service delivery versus training, and briefly the new Junior Doctor contract and the effects of Brexit on surgical training.

The purpose of the review is to attempt to apply strategic thinking and strategy development to improve the current state of surgical training given the current climate new trainees find themselves in. Strategic thinking and wicked issues are defined, and three umbrella suggestions to improve surgical training are explored. Whether these suggestions can be implemented with reference to different models of strategic decision making is discussed.

Finally, despite a new pilot scheme aimed at improving surgical house officer (SHO) surgical training, little change is offered to current trainees. The impact this has on surgical trainees is discussed and suggestions on how they can make the most of the current climate are made in this article.

## Introduction and background

Surgical training and its challenges

Ever since Dr. Halsted’s ambition to develop surgeons ‘of the highest type’, the need for high-quality surgical training has become embedded in modern surgical programmes [1]. The old adage ‘see one, do one, teach one’ simply does not exist in surgery any longer, and for good reasons. Surgical training is designed to produce surgeons that are competent in delivering the highest quality of surgical care to patients [2]. Surgical trainees are the future surgical leaders. However, there are a number of influences that negatively impact surgical training such that this outcome may be compromised unless change is made.

In the UK, there are multiple routes into specialist training and ultimately to consultancy [3]. Typically, the current training programme comprises of two years of foundation training then a competitive national selection process into a two year ‘core training’ programme. This is followed by a second competitive national selection process for ‘specialty training’ (ST3-8) which typically lasts six years but can last longer if any research, academia, fellowships or less-than-full-time training occurs due to parental leave or other reasons. The outcome is a Certificate of Completion of Training (CCT) allowing application to consultant posts.

Medical training has seen an overhaul in the past 20 years, and especially the last five years. Concerns particularly with surgical training are due to the very practical nature of the profession and the necessary ‘hands on’ experience in order to gain a CCT.

One of the biggest challenges for surgery in particular has been the European Working Time Directive (EWTD) in 1998, which was implemented in healthcare in 2009, limiting working hours to 48 hours per week calculated over a six month period. A number of issues have arisen. Changing from a ‘firm’ structure (a team of trainees working with an allocated consultant and caring for their patients only) to a shift-based rota (a team of trainees caring for patients of multiple consultants) has been detrimental to the trainer-trainee relationship fundamental to surgical training and continuity of care with multiple handovers [4]. This is compounded by a greater number of out-of-hours shifts to ensure service provision. A year later, due to concerns the EWTD severely impacted training with regards to reducing the number of hours available for supervised training, the Temple report was published [5]. It concluded that surgical training can be delivered within the confines of a 48 hours week but not if trainees have a major role in out-of-hours service, are poorly supervised or lack access to learning opportunities. Suggestions of a more consultant-led service, service reconfiguration, using more simulation in training and a Hospital-at-Night team to reduce service commitments were proposed; these suggestions have been implemented to various degrees.

The dichotomous issue of service provision versus dedicated training time has been an ongoing debate. Training is affected especially in the most junior years [6,7]. It is recognised by junior doctors that learning opportunities exist even whilst providing a service, but with ever-increasing pressure on the workforce due to increased admissions, an ageing population with increasingly complex medical co-morbidities and national targets across all areas of the National Health Service (NHS), the wards and clinics are busier than before. A subsequent impact on training is inevitable [8]. An increasing number of doctors are having a break from training after foundation years with burnout the most common reason [9]. The adverse impact on training is supported by the EWTD Taskforce (2014) which incorporated input from a range of stakeholders including medical professionals, the British medical Association (BMA), the Junior Doctors Committee, NHS employers, and patient representatives [10]. Again, there were recommendations to explore the possibility of separating service and training, and a greater flexibility with working patterns.

Since then, there has been the highly publicised, controversial new Junior Doctor contract imposed in 2016. Negotiations between the BMA, NHS Employers and the government began in 2013 and resulted in a number of episodes of strike action by the medical profession. Although the new contract creates some new and welcomed safeguards to rota design and safeguards working hours, it also applies further constraints to the rota to ensure they are ‘compliant’; there are 16 targets that must be satisfied for a rota to be deemed compliant [11]. This results in inflexibility and impacts training.

The theme that training could be delivered if training opportunities are recognised and protected is not realised in practice. The most recent General Medical Council (GMC) national survey (2017) highlights that although satisfaction with teaching and experience in post remains high, half of doctors work beyond expectations on a weekly basis, and almost a third of doctors have had training opportunities affected by rota gaps [12]. Similar results were found in each of the three previous Joint Committee on Surgical Training (JCST) annual survey reports for core trainees [13]. Since the implementation of the new doctor contract, social media has continued to be a platform on which trainees voice their concerns; #mindtherotagap remains a leading hashtag on Twitter [14].

Finally, the UK has voted to leave the European Union following a referendum. Certainly, a large proportion of NHS staff trained outside the UK; 40% of surgeons obtained their primary medical qualification outside the UK [12]. There are concerns further rota gaps will continue to arise due to European doctors leaving the UK after Brexit, which will only exacerbate the current issues surrounding surgical training in the UK [15]. Although laws regarding the 48 hour week and rest periods are enshrined in the Junior Doctor contract and will remain post-Brexit, there are some legal court rulings regarding the EWTD for which it is unclear if they will remain. The Position Statement released by the Royal College of Surgeons (RCS) calls for protection of the workforce, education and training time for junior doctors, as well as further NHS funding, of course [16]. The RCS president suggests 3,000 hours of extra surgical training time will be available post-Brexit [17].

## Review

Strategic thinking and wicked issues - how do they apply to surgical training?

Strategic thinking has been defined many times over, albeit in a non-precise manner [18-22]. Strategic thinking is likely to incorporate a mix of the elements that have previously been discussed in the literature, and as such, I offer an amalgamation of a few of these. ‘Strategic’ can be likened to ‘of great importance’, and namely concerns a process of finite resource allocation, be this military, civil, business or public sectors [21]. The NHS has finite financial constraints. In this context, strategic thinking can be considered to have:

Plans - be this a master plan (incorporating the ‘helicopter’ view [23]; ‘whole brain’ model [24] or ‘systems’ thinking approach [25]); a plan of action (a future thinking and ‘hypothesis’ driven [20] approach that is creative, flexible and tests new concepts [26]); and a game plan (aspects of operational thinking including strategic formulation, planning and deployment that are not indistinct from strategic thinking).

Perspectives - best described by Mintzberg’s seven frameworks of ‘seeing’ - ahead, behind, above, below, beside, beyond and through [18]. Importantly, this also incorporates stakeholders’ perspectives at various levels within an organisation, and the internal and external environments which will impact strategy. The current stakeholders related to surgical training include: the surgical trainees themselves; the JCST, via Specialty Advisory Committees (SACs) for each surgical speciality, who provide quality indicators against a defined curriculum in order to assess surgical trainees’ progress; the Royal Colleges to which trainees have membership; the Local Education and Training Boards (LETBs) who employ the trainees; NHS Employers and the Department of Health and Social Care who help shape the NHS as a whole; and of course, the present and future surgical patients.

Purpose - the intentional active implementation of strategic thinking in order to create sustainable change [20].

In essence, strategic thinking involves creating purposeful plans for the future taking into account all stakeholders’ perspectives.

Wicked issues, in contrast to tame issues, have ten characteristics which define them [27]. They are uniquely ill-defined and difficult to understand, with no right or wrong solution, and fundamentally they cannot be solved. However, any attempt at a solution will subsequently and significantly result in new issues. Importantly, the albeit intentionally sufficient solution to a wicked problem depends upon the perspective with which we attempt to understand the problem. Table 1 suggests how surgical training may be considered a wicked issue in relation to their common characteristics. From the surgical trainee perspective, surgical training has suffered due to a number of key issues as previously discussed including EWTD, changing rotas, new contracts and a change in the assessment process. Although some elements of this are true, personal experience tells me that some trainers take the view that even if it has changed, there is always opportunity (for example, attending lists on days off) and it is the trainee’s responsibility to ensure they are trained - ‘good trainees get trained’. Given satisfaction within posts remains quite good, training issues may not be seen as much of a problem at all by some stakeholders.

**Table 1:** Why surgical training is a wicked problem. CCT: Certificate of Completion of Training, EWTD: European Working Time Directive, NHS: National Health Service

Characteristics of wicked problems	How does this apply to the wicked problem of improving surgical training? Examples in practice
There is no definitive formulation of a wicked problem	What constitutes ‘better surgical training’? Is it more supervision, more operating time, more complex cases, or more choice in sub-speciality?
Wicked problems have no stopping rule	The needs of surgical patients are changing, more operations are required, and technology has changed, demanding new surgical expertise.
Solutions to wicked problems are not true-or-false, but good-or-bad.	Currently, completion of surgical training is marked by gaining CCT; however, a number of trainees take on post-CCT fellowships to gain experience/ confidence, and a ‘Junior Consultant’ role has emerged. Surgeons continually learn throughout their careers.
There is no immediate and no ultimate test of a solution to a wicked problem	Testing whether introducing more simulation rather than training more Advanced Care Practitioners will improve surgical training is unclear.
Every solution to a wicked problem is a ‘one-shot operation’; because there is no opportunity to learn by trial-and-error, every attempt counts significantly	The NHS has a finite financial resource and must ‘get it right’ the first time. Surgeons’ lives (what specialty, where they live, training opportunities) will be affected.
Wicked problems do not have an enumerable (or exhaustively desirable) set of potential solutions, nor is there a well-described set of permissible operations that may be incorporated into the plan	Would increasing hours worked, changing assessments, avoiding gaps in rotas effectively result in improved surgical training? No clear solution exists.
Every wicked problem is essentially unique	Not all surgeons consider surgical training a problem at all.
Every wicked problem can be considered to be a symptom of another problem	EWTD, the new Junior Doctor contract, increased consultant-led care, changing patient expectations, exposure to surgery as students/ juniors: they all contribute to the current state of surgical training.
The existence of a discrepancy representing a wicked problem can be explained in numerous ways. The choice of explanation determines the nature of the problem’s resolution	There is no clear solution – will more doctors filling rota gaps mean surgical trainees have increased surgical exposure and subsequent improved surgical training? It might not.
The planner has no right to be wrong	With so much time and finances invested in training surgeons, and public dependence, there is no immunity to error for planners.

The purpose of applying strategic thinking to surgical training

Ensuring surgical training is designed to produce surgeons who are competent and confident in delivering the highest quality of surgical care to patients can be considered a wicked issue. In order to protect the training of surgeons of the future, change is needed now. If the training programmes continue in their current state, surgeons of the future may not be able to provide the highest quality surgical care. The circular issue of poor training opportunities due to problems such as rota inflexibility or gaps, and service commitments leads to poorer training opportunities, with the consequent effect of greater demands on a smaller workforce as fewer trainees apply for surgery, which will see a crisis in the not too distant future [12]. The number of training posts at core surgical level has also fallen for the past five years despite the increasing workload [28].

Given the major changes such as EWTD, new doctors contract, and the unknown of Brexit, all of which are changes that are persistent with no opportunity to reverse the decision, we must embrace them and ensure they work for the future. The purpose of this report is to attempt to apply strategic thinking and strategy development to improve the state of surgical training given the current climate new trainees find themselves in.

Strategic thinking and understanding the issue

The ‘Design School of Thought’ has been one of the most influential models of strategic thinking for its simplicity and logical approach - examine the internal and external environment, match the internal capabilities and external possibilities, generate and evaluate routes for strategic success followed by implementation of the chosen strategy [29,30]. Performing a SWOT (strengths, weakness, opportunities and threats) analysis is often the first step in the Design School and has been extensively reviewed [31,32]. An example of some of the factors that influence surgical training in the UK today can be seen in Table 2. The fact that surgeons are needed by a future NHS gives job security to trainees and some consolidation for the lack of control they have over the threats impacting surgical training, which are often chronic, political or related to the changing population and service requirements of the NHS. Performing a SWOT analysis serves well to provide a superficial overview of potential internal and external factors, which it is assumed that if these are matched, then an organisation, or in this case, surgical training will prosper. It is simple, intuitive and often quick to perform, and as such, is considered early when reviewing an organisation’s strategy. However, it produces long lists of vague and ‘equal’ broad terms that have little substance to develop an effective strategy [33]. It also suffers subjectivity which can result from a number of cognitive biases, which will in turn affect the validity of the model [34]. Specifically in relation to surgical training, what is an opportunity for one surgical trainee may indeed be a threat to another.

**Table 2:** SWOT (strengths, weaknesses, opportunties, threats) analysis of current surgical training. ISCP: Intercollegiate Surgical Curriculum Programme, FYs: Foundation Year Doctors, NHS: National Health Service

Strengths	Weakness	Opportunities	Threats
Need for surgeons of the future – we must be trained.	Poor exposure to surgical training at junior levels (medical student, FYs) to make informed choice.	New run-through programs for surgery – job security for trainees at an earlier stage.	Political climate – Brexit, NHS secretary, doctor’s contract -> no control over these factors.
Clear competence outcome requirements on ISCP.	‘Tick box’ exercise of assessments for progression.	Change to consultant-led delivery of care – more time to train juniors.	Increased demand on NHS service with need for ‘quick’ turn around for surgical patients.
Wide variety of surgical specialities to accommodate all interests.	Lack of protected time in job plans for trainers.	Additional roles of extended surgical team may allow reduced service commitments for surgical trainees.	Winter pressures resulting in cancelled elective lists and longer waiting lists.
Inherently resilient workforce.	Lack of dedicated ‘lumps and bumps’ list for juniors: lack of protected training lists.		
	Poor recruitment of women into surgery despite changing health professional demographics.		

However, surgical training is inherently complex and a SWOT analysis simply does not go far enough. Improvements to a SWOT analysis would be to investigate or validate each point with clear examples that could be addressed strategically, and thus provide some meaningful output. It has been suggested that weighting and subsequently prioritising lists would be helpful [33]. Fundamentally though, SWOT analysis doesn’t consider all the vital aspects that are needed for an effective strategy. While PESTLE (political, economic, social, technological, legal and environmental) analysis does include some of these, again not all factors are incorporated and indeed, strategy development is more than ‘internal’ and ‘external’ factors, especially in a complex and constantly changing environment. An example of some of the factors that influence surgical training in the UK today from an NHS, patient and surgeon perspective using the PESTLE approach can be seen in Tables 3-5. Multiple adapted and new models have been used and explored [32]. Finally, while other schools of thought are discussed later, it is worth noting that strategy development can be considered to be multi-dimensional [35]. This model relates to aspects of surgical training: NHS employers, the colleges and the JCST have relative command over the training post numbers, content and delivery, and quality assurance, respectively; the planning dimension directly relates to workforce planning and number of posts available in each LETB; subtle changes to ISCP competency based-outcomes necessary for progression could be considered incremental processes; the new doctors’ contract is evidence of political processes that exist; a resistance to change from trainees regarding a ‘firm’ structure could be considered within the cultural dimension; but the necessity to change from a ‘firm’ structure due to EWTD has been enforced choice.

**Table 3:** PESTLE (political, economical, social, technological, legal, environmental) analysis of current surgical training from the NHS perspective. NHS: National Health Service, EWTD: European Working Time Directive, ISCP: Intercollegiate Surgical Curriculum Programme, A+E: Accident and Emergency, IT: Information Technology, Lap Trainer: Laparoscopic Trainer

Political	Economical	Social	Technological	Legal	Environmental
Current and future Health Secretary and political party – determines direction and funding.	Funding of NHS; impact of recession and economic recovery. Efficiency savings.	Growing population, with more A+E presentations and admissions.	IT systems – not linked up across the NHS and need for repeated training on similar systems.	The EWTD and its implications on limiting the hours in the working week.	Efficiency savings in the core of the NHS nowadays. Sometimes particular equipment isn’t available, or they have changed for cheaper alternatives (eg sutures).
Brexit and the unknown. Strong feeling for free healthcare at the point of need by all stakeholders.	Cost-neutral contract: no pay increases, changes to pay schedule – may not encourage juniors to apply for surgery.	Population demographics: older, obese, more complex, riskier surgery – perhaps fewer training opportunities.	Simulation training including lap trainers/ boxes/ virtual reality – able to use these but often restricted with office hours. Virtual reality headsets on the increase but costly.	The new Junior Doctor contract and its implications regarding shift work and training opportunities.	IT systems in some hospitals which are now ‘paper free’.
EWTD and more recently ‘seven day’ NHS and requirement for change in shift patterns.			Impact of the internet – YouTube videos detailing operations; change in learning style		
Responsibility for trainee learning – does it lie with regulators (ISCP, deanery), trainer or trainee?					

**Table 4:** PESTLE (political, economical, social, technological, legal, environmental) analysis of current surgical training from a patient's perspective. CCT: Certificate of Completion of Training

Political	Economical	Social	Technological	Legal	Environmental
Winter pressures, cancellation of elective cases in order to maintain acute services. Most learning done during day shifts and elective cases.	Cost of training overall and need to ensure value for money for public.	Public perception and expectations of surgeons in training and post-CCT. Can patients tell the difference? Should they expect a difference? There is a drive for consultant-led care.	The introduction of virtual fracture clinics – could this be extended to other clinics?	Cancelled patients due to ‘winter pressures’ – what impact could this have if harm came to the patient, and what are the legal implications for a service that ‘let down’ a patient? The impact is that each operating list is pressured and reduced trainee operating time.	Virtual surgical clinics – could these be the future?
		Impact of strikes during doctor contract issues on perception.	With increasing availability of radiological imaging, more scans are being requested and these often help plan surgery.		

**Table 5:** PESTLE (political, economical, social, technological, legal, environmental) analysis of current surgical training from a surgeon's perspective.

Political	Economical	Social	Technological	Legal	Environmental
Doctors’ employment contract.	Thousands of pounds for exams, courses and conferences ‘hidden costs’.	Media portrayal of doctors and surgeons. Negative impact with ‘scandals’.	Change in operative approach – increase in laparoscopic surgery. Trainees may worry about ability to perform open surgery too.	Change from ‘experiential’ to ‘competence’ training and need for ISCP and ARCP annual appraisals. How is competence measured?	Videos and online distance learning rather than face-to-face courses increasing in popularity. Some courses have reduced in time as more of the course becomes ‘pre-course’ material.
Impact of shift work on training opportunities.	Regional differences in availability of a study budget for external courses to aid with training; often difficult process with various forms to complete.	Uncertainty during national application process.		Use of portfolio reflections regarding incidents used against trainees in court – how can doctors effectively reflect on difficulties and how to improve, as is expected?	
Surgeon reported data – could influence likelihood of trainee being allowed to perform a particular operation, especially if more complex.		Need for trainees to rotate posts – where to live and family commitments.			

Three key problem areas, specifically in relation to surgical training, can be drawn from the SWOT/ PESTLE analysis and suggestions for improvement are now discussed.

The trainer-trainee relationship

Current surgical training has seen a vast change in number and range of operations performed, especially at SHO level [36]. Gone are the days of SHO-led ‘lumps and bumps’ lists in favour of consultant-led care. Whilst this should lead to more opportunities to be assessed by consultants and have directed constructive feedback, sheer volume and ‘need to get through an operating list’ impacts this. Each level has its own pressures. From personal experience, trainees spend less clinical time with the same consultant, or team of consultants, due to other work commitments and rota demands. The knock-on influence this has is that trainers do not know their trainees as well as they would like, and vice versa, and may explain, in part, why surgical trainees have less operative exposure [2].

Ultimately, there needs to be a change in the trainer-trainee relationship, which, although is unlikely to ever return to days pre-EWTD, is necessary. There are a number of ways to achieve this.

There needs to be change at multiple levels including those at the level of the trust, at the level of the JCST and at the level of NHS employers. Changing the rota such that trainees spend an elective block, which is predominantly training focussed, with one trainee per supervised consultant session, is likely to help. This elective block should be free from on call commitments and would provide the consistent trainer-trainee interactions necessary for trainer-trainee rapport. The JCST ought to consider changing the ‘Quality Indicators’ (a marker of quality for each rotation through which a trainee rotates) from there is an ‘expectation’ to attend consultant-supervised sessions to a ‘necessary’ to attend consultant-supervised sessions. Finally, trainers need access to resources in the form of contractually-protected time with trainees, focussed training with the ISCP, and remuneration.

Training and service requirements

Rota gaps, as previously discussed, are increasing and negatively impact the training-service relationship. A third of trainees and trainers believe rota gaps impact training opportunities [9]. Recently, the ‘Extended Surgical Team (EST)’ composed of Surgical Care Practitioners, Surgical First Assistants and Physician Associates to name a few, who are non-medical practitioners involved in pre-, intra- and postoperative care under the supervision of a consultant, has been developed [37]. The premise is that this team may help provide service needs allowing surgical trainees more time for training [28]. Concerns exist amongst trainees that these new roles may further dilute their own surgical exposure especially regarding clinic and theatre time despite reassurance this will not be the case [28,37]. However, compared to surgeons in training that rotate around multiple hospitals within an LETB to gain a wide range of experience, the EST is a constant presence in one speciality. This will provide continuity of care for patients and has other benefits such as allowing a quick and smooth ‘settling in’ period when training surgeons have rotated to a new hospital. Consultants also favour this level of continuity and stability the EST provides and is synonymous with the trainee-trainer relationship that is the envy of training surgeons [28]. Other concerns regarding quality assurance, regulation, the ESTs’ own training and progression needs also need to be considered before the wide implementation of this new team.

Although increasing the number of surgical trainees in post would be an ‘ideal’ solution, the implementation of the EST may be a viable alternative. It is currently recommended by the Royal College of Surgeons [28].

Competency-based training

With a ‘time-limited’ training programme is the inevitable development of a competency-based curricula to ensure trainees are appropriately trained to the standard required for CCT. This also allows for transparency in surgical training, which is fundamental for evidence-based learning. Competence-based training was introduced in the UK in 2007 [38]. Assessment of competence is multifaceted and uses a number of different mediums, including workplace-based assessments (WBAs) for different settings, examinations, and supervisor meetings. This culminates in a portfolio (the Intercollegiate Surgical Curriculum Programme (ISCP), paid for annually by surgical trainees themselves) of assessments which is reviewed at an Annual Report of Competency Progression (ARCP) meeting [39]. A number of criteria for progression are often detailed at the start of a training programme, which unfortunately can lead to trainees viewing these as ‘tick boxes’ and can detract from the original educational purpose of the assessments [40]. Other issues with WBAs include the true extent to which the assessments provide useful feedback to trainees, a lack of engagement, a lack of validity, an increased administrative burden on the part of the trainer and trainee and on whom responsibility for trainee learning falls (the trainee, the trainer, the supervisors or the programme co-ordinators) for obtaining these WBAs [40]. This is on top of the frustration trainees feel when chasing assessments to be completed and trainers finding the time to provide satisfactory feedback to the benefit of the trainee. However, my main concern is the assumption that completion of the required number and level of WBAs equals competence.

No clear assessment process is likely to exist but ultimately the purpose of assessments is to ensure a CCT can be awarded, and thus the surgeon is competent and confident in delivering the highest quality of surgical care to patients. A recent report by the Royal College of Surgeons suggests a refined assessment tool such as the ‘Entrustable Professional Activity’ may be beneficial [2]. Whilst there may be some benefit, surgical training is already saturated with multiple assessments.

Surgical training and strategy in practice

The above proposals for change follow the normative model of decision making (or Bailey and Johnson’s ‘Planning’ strategy development process, or Mintzberg’s ‘Design or Planning’ Schools of Thought). In much the same way upon which clinical audits are based, a problem is identified and understood, and often compared against a set of standards (the competency-based curricula as defined by ISCP, as well as the expected outcomes for CCT), with a range of solutions developed that will satisfy the problem, one or multiple of which are implemented and further re-evaluated [41]. As we have seen, problems with surgical training are not isolated. They are complex - wicked, in fact - and influenced by many factors; consequently, more than one ‘solution’ is likely to be required, and in not such a linear pathway. Any such solution is likely to be complex, have multiple downstream effects, some of which may be unknown and unforeseen. In this current climate, effective strategic decision making to improve surgical training will have to be dynamic, flexible and may need to make more dramatic and proactive changes to curtail the current and future challenges.

The above decision making processes very easily relate to Mintzberg's view of strategy as a ‘plan’; an intended and, hopefully, realised strategic decision [42]. Although these three proposals above may improve surgical training, the key to influencing such changes will be an increased awareness and engagement from all stakeholders including the JCST, NHS employers, the colleges and the surgical trainees. Each stakeholder has their own agenda, and as discussed earlier, surgical training may not even actually be seen as an issue by some stakeholders. In this context, surgical trainees, who arguably are the main drivers of change to their own training, would find it beneficial to unite and engage more thoroughly with membership groups such as the Association for Surgeons in Training and College representatives, in order to apply pressure to the real directors of change and decision makers such as the JCST, LETBs and NHS employers. They could go as far as to complete the same assessments for competency on ISCP, or not complete certain assessments, to highlight the deficiencies in the current system. With the change in range and exposure to particular operations and difficulty in obtaining ‘competency’ for such a procedure, this is in fact being realised to some extent unintentionally. Although this of course wouldn’t be done deliberately, in this sense the strategy can be, at least in part, a ‘ploy’ too. Strategy as ‘pattern’ is evident from the fact that the RCS and Health Education England (HEE) have recently developed a pilot scheme which precisely aims to improve the quality and quantity of surgical training at SHO level, which takes into account aspects of the three above proposals [2,43]. Specialist training has not been addressed. The Shape of Training Review in essence is a review of previous strategies and initiatives that in part forms the basis of this new pilot scheme [44]. Strategy as a ‘position’ applied to surgical training is initially unclear. However, surgical trainees in the UK can only be trained ultimately through one training pathway, thus creating a niche within which trainees find themselves. Finally, given the repeated findings of the annual GMC national trainee surveys (such as missed training opportunities due to service commitments), the collective mind of surgical trainees’ represents strategy as ‘perspective’ [45].

The process of making strategic decisions regarding surgical training may be improved by using a different model to that used thus far; no more surveys, reviews and reports which ‘inform’ the next stage of the strategic plan. ‘Prescriptive’ decision making has been tried and tested. A number of Mintzberg’s Schools of Thought are not solely appropriate at this moment of time [30]. The Cognitive School is less applicable beyond the conceptualisation stage, which is unsustainable for surgical training; the Power School ought not be accepted as there can be no compromise or concessions on the high standard of surgical care that needs to be maintained for patients, and consequently the high standard of surgical training - however, arguably, politics does play a major role in the NHS, though this is less than ideal; the Cultural School is inclusive and draws on values and beliefs of the stakeholders but may lead to minimal changes to the status quo of surgical training; whilst the Environmental School could offer key strategic changes to external factors that will impact training such as Brexit, this is not appropriate for sustainable workforce planning or ensuring training needs are met over the years it takes to be trained. No one view is complete by itself and components of each School of Thought may be more or less useful at various times during an organisation’s development. However, I suggest the Learner School would be most appropriate at this current time. Many lessons can be learned from the recent and significant aforementioned challenges facing surgical training, which have been discussed repeatedly in multiple reports. Some of the most recent solutions have been insufficient to effect real change in creating a sustainable high-quality surgical workforce for the future. Therefore, the Learner School of thought may be most useful before there is a crisis in surgical training.

How can trainees embrace the challenges with surgical training?

As the ARCP deadline approaches, all surgical trainees will be ensuring their competency has been adequately and rigorously assessed in order to ensure a smooth progression to the next stage. Whilst there are proposals and pilots in place to remedy issues with surgical training for some new SHOs, I will progress to registrar level and face the same issues detailed above, but with a clearer perspective. It is unlikely sweeping changes will affect current SHO or registrar surgical training, but there are ways of maximising potential learning opportunities in relation to the three identified key strategic areas to ensure current trainees reach requirement for CCT in the years to come.

Quickly building a solid rapport with my trainers is important. This will start with seeking out trainers before starting new posts, and more importantly, developing a professional development plan as soon as possible in order to formalise any learning agreements. Creating short and long term achievable goals, within the confines of other service commitments, will be fundamental to succeeding. Knowing where and how to achieve these (most useful and appropriate clinics/ theatre for the corresponding level of training) will be the most vital, and is likely to depend on embracing the EST. Another important factor will be to continue to embrace the ISCP, but if possible in a more structured fashion. Whilst current WBAs are produced ad-hoc after an interesting or difficult case, perhaps a more formalised session once or twice a month will ensure the avoidance of ‘see and do more’ feedback and allow a regular meeting with the trainer, which will further foster a better trainee-trainer relationship.

What this means for the future of surgical trainees

For all stakeholders, success will be measured on paper by attainment of CCT but in practice by producing surgeons who are competent in delivering the highest quality of surgical care to patients. For surgical trainees, having the confidence as well as competence to cope with the changing demands of the future may only emerge as an issue in time. The development of more fellowships and sub-consultant roles would be a symptom of this and should not be considered a solution [46,47].

There have been, and are, ongoing major challenges and changes to surgical training, with the full effects of previous strategies only being realised in recent years. Whilst past and current strategic thinking has followed a more normative method, with pilots such as Improving Surgical Training (IST) aimed at alleviating these recognised issues, I believe these will not go far enough given the changing demographics of the surgical patient, the changing environment in which surgeons work and the continued demand for the highest of standards in a politically less stable world. The intended strategy will not be realised, or only partly realised, and certainly isn’t complete as it doesn’t address current trainee needs.

Whilst the focus of this report regarding surgical training has focussed on surgical trainees in the midst of training, training actually begins in medical school. Exposure to anatomy, basic surgical skills and the theatre environment is often overlooked in medical school, and these subsequently impact the satisfaction with surgical teaching amongst medical students in the UK and whether they apply for further surgical training later in their careers [48]. There is a strong argument that strategic thinking and development should extend beyond the current surgical trainee’s training and to future potential surgical trainees.

## Conclusions

A different model of strategic thinking needs to be applied to surgical training in the UK. Current models based on prescriptive/ normative thinking, with successive reports and agendas for change have not produced change that is adequate or sustainable. Current surgical training is being adversely affected by major political issues such as the EWTD, service provision and the Junior Doctor contract. Major future challenges such as the impact of Brexit are looming and a new approach is needed.

Whilst there is a good understanding of the important issues that are and will affect surgical training, there appears to be no clear, sustainable strategy for how best to develop surgical training in the face of a changing and challenging climate, yet maintaining the necessary high-quality standards surgeons and patients expect. It is suggested that re-invigorating the trainer-trainee relationship, embracing the extending surgical team, and refining of competence-based assessments may be key.

## References

[1] Cameron J (1997). William Stewart Halsted: our surgical heritage. Ann Surg.

[2] Royal College of Surgeons (2018). Royal College of Surgeons: improving surgical training. London: Royal College of Surgeons England.

[3] Sheikh Z (2017). Increased working hours, simulation technology or competency based progression? What is the solution for surgical training?. Surgery (Oxford).

[4] (2019). British Medical Association: Safe handover: safe patients. https://www.bma.org.uk/-/media/files/pdfs/practical%20advice%20at%20work/contracts/safe%20handover%20safe%20patients.pdf.

[5] Temple J (2018). Time for training: a review of the impact of the European Working Time Directive on the quality of training. https://www.londonmedicine.ac.uk/wp-content/uploads/2016/11/Temple%20Report_Time%20for%20Training_%20May%202010.pdf.

[6] Eardley I (2018). The Royal College of Surgeons: the shape of training. The Bulletin of the Royal College of Surgeons of England.

[7] Lewington K (2012). Changes to medical education over the past 20 years. Br Med J.

[8] Rimmer A (2014). Trainees call for implementation of Shape of Training review to be paused. Br Med J.

[9] General Medical Council (2018). General Medical Council: the state of medical education and practice in the UK report. General Medical Council.

[10] (2018). The Royal College of Surgeons: working time directive taskforce. London: RCS Publications.

[11] British Medical Association (2018). British Medical Association: rostering guidance. British Medical Association.

[12] General Medical Council (2018). General Medical Council: national training surveys result. Survey Reports. General Medical Council.

[13] (2018). Joint Committee on Surgical Training: third annual report of the JCST trainee survey. https://www.jcst.org/quality-assurance/trainee-survey/.

[14] Gulland A (2017). Sixty seconds on . . . mind the rota gap. Br Med J.

[15] Torjesen I (2017). Four in 10 European doctors may leave UK after Brexit vote, BMA survey finds. Br Med J.

[16] Royal College of Surgeons (2018). Royal College of Surgeons: 2016 position statement: making the best of Brexit for the NHS. Accessed.

[17] Bodkin H (2018). Brexit will make the NHS safer, top surgeon says. The Telegraph. 2016. Accessed.

[18] Mintzberg H (2003). Strategic thinking as 'seeing'. Developing Strategic Thought: A Collection of the Best Thinking on Business Strategy.

[19] Young L (2016). Developing strategic thinking. AAJ.

[20] Liedtka J (1998). Strategic thinking: can it be taught?. Long Range Plann.

[21] Nickols F (2018). Strategy, strategic management, strategic planning and strategic thinking. https://www.nickols.us/strategy_etc.pdf.

[22] Pattinson S (2016). Strategic thinking: intelligent opportunism and emergent strategy — the case of strategic engineering services. Int J Enterpren Innovat Manag.

[23] Hanford P (1995). Developing director and executive competencies in strategic thinking. Developing strategic thought: Reinventing the art of direction-giving.

[24] Herrmann N, Herrmann-Nehdi A (1996). The Whole Brain Business Book. The Whole Brain Business Book.

[25] Bonn I (2001). Developing strategic thinking as a core competency. Manage Decis.

[26] Mintzberg H (2018). Harvard Business Review: the fall and rise of strategic planning. Harvard Business Review.

[27] Rittel H, Webber M (1973). Dilemmas in a general theory of planning. Policy Sci.

[28] Royal College of Surgeons (2018). Royal College of Surgeons: a question of balance. London: Royal College of Surgeons.

[29] Mintzberg H (1990). The design school: reconsidering the basic premises of strategic management. Strategic Manage J.

[30] Mintzberg H, Ahlstrand B, Lampel J (2009). Strategy Safari: A Guided Tour Through The Wilds of Strategic Managament. https://books.google.com/books?hl=en&lr=&id=zOMIuP4ZS5gC&oi=fnd&pg=PA5&dq=Strategy+Safari&ots=2aRhtUiyhV&sig=QEFRnJa2pM3_TMss6e5VuKICBSc#v=onepage&q=Strategy%20Safari&f=false.

[31] Sarbah A, Otu-Nyarko D (2014). An overview of the design school of strategic management (strategy formulation as a process of conception). Open J Bus Manage.

[32] Gurel E, Tat M (2017). SWOT analysis: a theoretical review. J Int Soc Res.

[33] Hill T, Westbrook R (1997). SWOT analysis: It's time for a product recall. Long Range Plann.

[34] Montibeller G, von Winterfeldt D (2015). Cognitive and motivational biases in decision and risk analysis. Risk Anal.

[35] Bailey A, Johnson G, Daniels K (2000). Validation of a multi-dimensional measure of strategy development processes. Brit J Manage.

[36] Blencowe N, Parsons B, Hollowood A (2011). Effects of changing work patterns on general surgical training over the last decade. Postgrad Med J.

[37] Bruce C, Bruce I, Williams L (2006). The impact of surgical care practitioners on surgical training. J R Soc Med.

[38] Shalhoub J, Santos C, Bussey M, Eardley I, Allum W (2015). A descriptive analysis of the use of workplace-based assessments in UK surgical training. J Surg Educ.

[39] Kabir S, Kabir S (2014). Competency based surgical training. Int Surg J.

[40] Swayamprakasam A, Segaran A, Allery L (2014). Work-based assessments: making the transition from participation to engagement. JRSM Open.

[41] Teale M (2003). Management Decision Making.

[42] Mintzberg H (1987). The strategy concept I: five Ps for strategy. Calif Manage Rev.

[43] Royal College of Surgeons (2018). Royal College of Surgeons: improving surgical training. London: Royal College of Surgeons.

[44] Greenaway D (2018). Shape of training: securing the future of excellent patient care. London.

[45] General Medical Council (2018). General Medical Council: training pathways: analysis of the transition from the foundation programme to the next stage of training. General Medical Council.

[46] Rimmer A (2014). Surgeons are less satisfied with training than other doctors, GMC survey finds. Brit Med J.

[47] Rimmer A (2014). What lies ahead for the subconsultant grade?. Brit Med J.

[48] Lee M, Drake T, Malik T, O’Connor T, Chebbout R, Daoub A, Wild J (2016). Has the bachelor of surgery left medical school?—a national undergraduate assessment. J Surg Educ.

